# Cell Lysis Based on an Oscillating Microbubble Array

**DOI:** 10.3390/mi11030288

**Published:** 2020-03-10

**Authors:** Xiufang Liu, Jinyuan Li, Liangyu Zhang, Xiaowei Huang, Umar Farooq, Na Pang, Wei Zhou, Lin Qi, Lisheng Xu, Lili Niu, Long Meng

**Affiliations:** 1 College of Medicine and Biological Information Engineering, Northeastern University, 195 Innovation Road, Shenyang 110016, China; 2 Paul C. Lauterbur Research Center for Biomedical Imaging, Institute of Biomedical and Health Engineering, Shenzhen Institutes of Advanced Technology, Chinese Academy of Sciences, 1068 Xueyuan Avenue, Shenzhen 518055, China; 3 Guangdong-Hong Kong-Macao Greater Bay Area Center for Brain Science and Brain-Inspired Intelligence, Guangzhou 510515, China

**Keywords:** cell lysis, ultrasound bioeffects, stable cavitation, shear stress

## Abstract

Cell lysis is a process of breaking cell membranes to release intracellular substances such as DNA, RNA, protein, or organelles from a cell. The detection of DNA, RNA, or protein from the lysed cells is of importance for cancer diagnostics and drug screening. In this study, we develop a microbubble array that enables the realization of multiple cell lysis induced by the shear stress resulting from the individual oscillating microbubbles. The oscillating microbubbles in the channel have similar vibration amplitudes, and the intracellular substances can be released from the individual cells efficiently. Moreover, the efficiency of cell lysis increases with increments of input voltage and sonication time. By means of DNA agarose-gel electrophoresis, a sufficient extraction amount of DNA released from the lysed cells can be detected, and there is no significant difference in lysis efficiency when compared to cell lysis achieved using commercial kits. With the advantages of the simple manufacturing process, low cost, high efficiency, and high speed, this device can serve as an efficient and versatile tool for the single-cell sequencing of cell biology research, disease diagnosis, and stem cell therapy.

## 1. Introduction

With the popularization of separation and purification of cell contents in disease diagnosis, gene therapy, tissue engineering, and other related experiments, cell lysis has attracted increasing attention in recent years [1,2,3]. Cell lysis is a technique to break the cell membrane, upon which cellular DNA, RNA, or protein can be released. Developing an efficient cell lysis strategy is crucial for separating and purifying intracellular components [3,4,5]. Current methods of cell lysis can be categorized based on biology [6,7] and chemical [8,9,10] or physical methods [11,12,13]. The biological and chemical methods can prevent mechanical damage to the intracellular components, which are widely used to extract sensitive cell contents from most kinds of cells [14,15].

Physical methods, such as thermal lysis, osmotic shock lysis, electric lysis, and acoustic lysis, can lyse the cell membrane in a non-contact manner. Thermal lysis is mainly based on successive freezing and thawing cycles [16]. Johnson et al. have efficiently separated highly expressed recombinant proteins from *E. coli* using freezing and thawing cycles. Osmotic shock induces cell lysis by causing the cells to swell and burst [11]. This results from the higher concentration of intracellular rather than surrounding solution, making it suitable for fragile membrane structure. Electrical field-induced cell lysis is highly dependent on the input voltage and pulse width. Han et al. have developed a capillary electrophoresis technique to rapidly lyse an adherent mammalian cell using a single pulse [17]. To achieve cell lysis induced using an electric field, a high voltage is required. High voltage, however, may lead to significant temperature elevation, resulting in protein inactivation.

Acoustic lysis enables the destruction of cell membranes by ultrasonic cavitation, particularly in the presence of microbubbles. According to different dynamic behaviors of bubbles, cavitation effect can be divided into inertial cavitation (formerly called transient cavitation) and stable cavitation (also called non-inertial cavitation) [18]. Compared to inertial cavitation, the acoustic pressure required for stable cavitation is not too high, and the process of stable cavitation is more controllable [19,20,21,22]. However, cavitation seems to be a random process, and it is difficult to break the cells completely. Therefore, a desired device based on acoustic cavitation should realize cell lysis in a controllable manner with a low power consumption.

With the development of micro- and nano-fabrication processes, microfluidic devices have been applied to biology for many years [23,24,25,26]. A microfluidic device has several distinct advantages, such as miniaturization, rapid analysis, ease of integration, and low energy consumption. Cell lysis based on acoustic microfluidics has received increasing attention. Marentis et al. designed and fabricated an acoustic device with a piezoelectric transducer [5]. Using a sinusoidal source in the 360-MHz range, they achieved the successful lysis of an HL-60 cell with 80% lysis efficiency. However, the cell lysis efficiency was not very high. Tandiono et al. demonstrated that the rod-shaped *E. coli* bacteria can be disrupted into small fragments in less than 0.4 s [27]. In their study, a gas pressure controller was utilized to import gas to the polydimethylsiloxane (PDMS) channel, and the air bubble was generated randomly. The cavitation of the air bubble induced by ultrasound was to achieve bacteria lysis. The stability of the cell lysis outcome is limited by the nonuniform distribution of the air bubbles.

The concept of oscillating microbubbles for cellular lysis has been demonstrated 17 years ago in Marmottant, et al. study [28]. The bubble radius of 10–100 um (controlled by the amount of air with syringe injection) was attached by capillary forces to the walls of a quartz cuvette. The mechanism for cell lysis mainly depends on shear force resulting from acoustic streaming. In this study, we established an efficient method for lysing cells in a microfluidic chip using an oscillating microbubble array. The cell lysis was mainly influenced by the shear stress generated by the oscillating microbubbles. As all microbubbles are produced with similar oscillation amplitude induced by a single frequency excitation (107 kHz), a high efficiency of the cell lysis can be achieved by the acoustic lysis device. This method can be used for single-cell analysis, microfluidic biological detection and diagnostic platform. This approach can be used for single-cell analysis and diagnostic platform.

## 2. Materials and Methods

### 2.1. Fabrication of the Microchip and Acoustic Field

**Figure 1:**
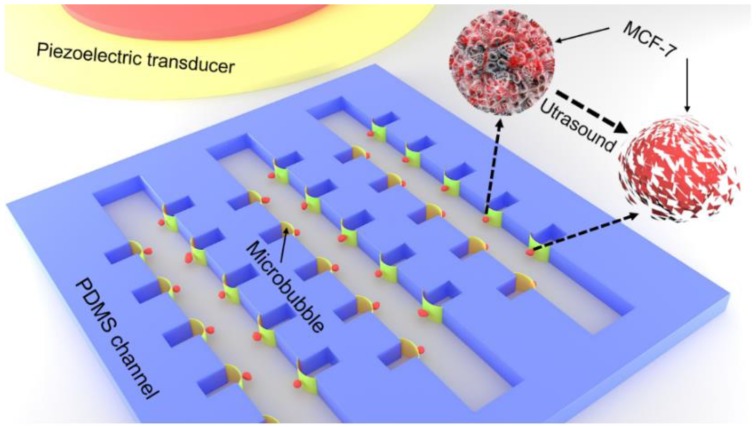
Schematic of experiment setup. The polydimethylsiloxane (PDMS) channel and piezoelectric ceramic transducer (PZT) were placed on the surface of the glass substrate. The PZT was employed to excite microbubble oscillation.

A microfluidic channel was fabricated using the standard replica molding technique [20,29,30]. The geometry of the device is shown in Figure 1. The device was mainly composed of two parts: a PDMS channel and a piezoelectric ceramic transducer (PZT). The fabrication process of the PDMS channel: After removing the residual impurities on the surface of the silicon wafer with alcohol, pickling and water, the silicon wafer was spin-coated by photoresist. Then, negative photoresist (SU-8 50, Microlithography Chemical Corp., Newton, MA, USA) was put onto silicon wafers to form a thin film at 550 rpm for 2 min. The adhesion between the silicon wafer and photoresist could be enhanced by placing the silicon wafer onto a horizontal heating plate at 60 °C for 4 min and 90 °C for 5 min. The photoresist was then exposed to an ultraviolet light source at a dose of 600 cJ/cm^2^ for a duration of 20 s and developed in the photoresist developer. The PDMS prepolymer (Glue A) and curing agent (Glue B, Sylgard 184, Dow Corning, Midland, MI, USA) were mixed at a ratio of 10:1. The mixture was poured onto the silicon template, vacuum degassing, and baked at 80 °C for 30 min. The cured PDMS was stripped from the silicon template and a puncher (Harris, Uni-Core, Jed Pella, Inc., Redding, CA, USA) was used to create the entry and exit of the microchannel. Finally, the microchannel was bonded to the glass substrate by the plasma treatment. The PZT transducer was placed adjacent to the PDMS microchannel on the same substrate with ultrasound coupling agent (Guang Gong, Guang Dong, China), to ensure that the acoustic energy could be coupled to the substrate efficiently. The rectangular PDMS channels array with the uniform size was fabricated at the sidewall. The PDMS channels could generate an array of monodisperse microbubbles with the same oscillation by ultrasound, ensuring cell lysis with a high efficiency. To ensure the free oscillation, the depth of the microchannel was deigned to be 50 μm. The width of the rectangular channel was approximately 40.8 μm, respectively, measured by a step profiler (XP1, MTS, San Jose, CA, USA). The transducer was composed of PZT-4 ceramic with a diameter of 26 mm and, in our experiment, was operated in the thickness vibration mode.

Surface tension is a key property of liquid–air interfaces. As an important factor in the formation of bubbles at the gas–liquid phase interface, surface tension can be defined as the contraction force acting on any part of the liquid surface on a straight line per unit length. Due to the hydrophobic treatment of PDMS channel in advance, the presence of surface tension makes the bubble formation easy in the PDMS channel when the liquid flows through. According to the ordinary Young–Laplace equation for a spherical interface, we know that surface tension is related to liquid–gas pressure difference and PDMS channel size [31]. Moreover, from Kumar and Kuloor (1970) study, we obtain that orifice size and material-dependent surface wettability as equipment variables affect bubble formation [32]. The resonant frequency of the microbubble was approximately 107 kHz and a more detailed calculation can be found in previous work [20]. The signal amplified by a power amplifier (100A400A, Amplifier Research, Souderton, PA, USA) was applied to PZT to excite the microbubble vibration.

We applied a laser Doppler vibrometer (LDV, UHF-120, Polytec, Berlin, Germany) to measure the acoustic pressure within the microchannel. The LDV was the propagation direction of acoustic waves and was utilized to measure the vibration of glass substrate in a non-contact manner. The LDV’s laser beam was positioned perpendicular to the surface of the glass substrate. As the surface of the glass substrate moved, the vibration amplitude could be obtained from the Doppler shift of the reflected laser beam frequency.

### 2.2. Measurement of Air Bubble Stable Cavitation and Deformation

The LDV was utilized to measure the frequency spectrum of the microbubble oscillation, acting as a passive cavitation detector (PCD) [33]. A laser beam with a tiny light spot (<2.5 μm) produced by the LDV was focused on the oscillating bubble surface to detect the Doppler-induced signals. If harmonic signals emitted by the oscillating microbubble could be detected, stable cavitation occurred. The deformation of the microbubble was captured by a high-speed charge-coupled device (CCD) camera (MC1310, Mikrotron, Lower schlesheim, Germany) with 1000 frames/s (exposure time: 2 ms; gain: 1; objective: 20×).

### 2.3. Cell Sample and Particle Preparation

The human breast cancer MCF-7 was obtained from the cell bank of the Chinese Academy of Science. Cells were cultured in Dulbecco’s Modified Eagle’s Medium (Gibco, Life Technologies, Carlsbad, CA, USA) and supplemented with 10% fetal bovine serum (FBS, Gibco, USA), 1% penicillin-streptomycin (penicillin 100 U/mL and streptomycin 100 mg/mL), and 1% glutamine. Cultures were maintained at 37 °C with humidity and 5% CO_2_. MCF-7 cells collected were centrifuged at 1000 rpm for 4 min in a centrifuge tube. After being washed with cold phosphate-buffered saline (PBS) twice, the cells were suspended in cold PBS for following experiments. The concentration of cells suspended in the cold PBS was adjusted to be approximately 2 × 10^6^ cells·mL^−1^. The 2 μm polystyrene particles (Sigma-Aldrich, St. Louis, MO, USA) were diluted in 0.1% Tween-80 DI water solution, preventing the adhering of samples to the substrate.

### 2.4. Cell Lysis in a Microfluidic System

The cell solution was injected into a channel via a syringe pump at a flow rate of 0.2 μL·min^−1^ (neMESYS, Cetoni, Korbussen, Germany). The chip was rinsed with DI water prior to the introduction of the cell solution. To monitor cell lysis, the MCF-7 cells were introduced at a density of 2 × 10^6^ cells·mL^−1^. Prior to the application of ultrasound, the cold PBS was supplemented with the fluorescent stain Calcein-AM (C1359MSDS, Sigma-Aldrich, USA) at a concentration of 6 μM and the dead stain propidium iodide (PI, P4864-10ML, Sigma-Aldrich, USA) at a concentration of 8 μM for 15 min.

The cell lysis efficiency was accessed by the fluorescence imaging method and was calculated with the following formula: Lysis efficiency (%) = Count_cell-PI_/Count_cell-Calcein-AM_ × 100% (Count_cell-PI_, red cells; Count_cell-Calcein-AM_, green cells). Each experiment was repeated five times, and fluorescence images were processed using the Image J software (8.0, National Institutes of Health, Bethesda, ML, USA).

### 2.5. DNA Gel Electrophoresis Study

After cell lysis, the sample solution in the channel was collected from the outlet with a clean tube. RNA contamination was averted using an RNase solution (10 mg/mL) for 10 min at 55 °C, and proteins were removed using proteinase K (10 mg/mL) for 3 min at 55 °C. Additionally, cellular debris was removed by centrifugation to obtain the purified DNA. Meanwhile, the genomic DNA in cells was also obtained with the chemical DNA extraction kit (D1700, Solarbio, Beijing, China). After that, 10 μL of the sample was mixed with 2 μL 6×loading buffer. Subsequently, it was resolved by electrophoresis in 1% agarose gel supplemented with 0.01% GelRed (4100, Biotium, Fremont, CA, USA), running for about 25 min at the voltage of 120 mV. Finally, the samples were visualized by UV light (Universal Hood II, Bio-Rad, San Diego, CA, USA).

### 2.6. Statistical Analysis

All experiments were performed five times. An independent sample t-test and analysis of variance (ANOVA) were implemented and employed for comparison between multiple groups by using the statistics software SPSS ver.12.0. The level of statistical significance was set at a *p* value < 0.05, and the level of high significance was set at a *p* value < 0.01.

## 3. Results and Discussion

### 3.1. Microstreaming, Shear Stress and Cell Trapped Induced by Oscillating Microbubble

**Figure 2:**
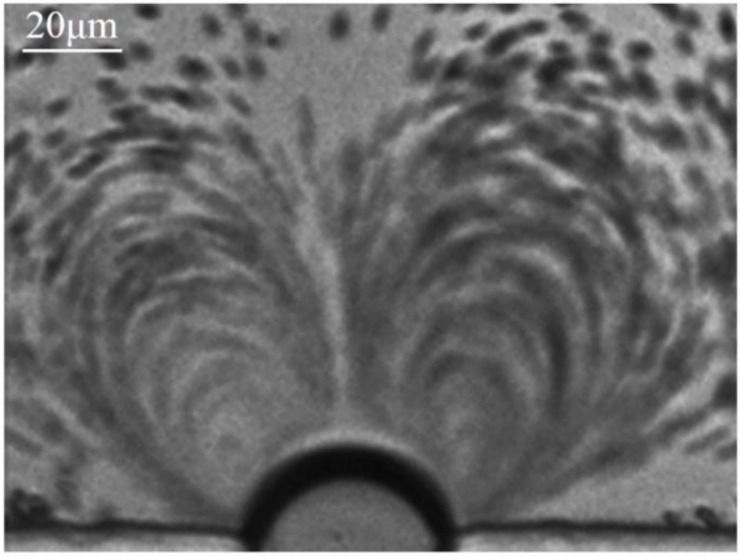
Optical field image of microstreaming generated by microbubble oscillation. When the PZT is excited, the 2 μm polystyrene particles around the microbubble is driven to follow the microstreaming.

Experimentally, a series of PZT transducers with a resonant frequency of 100–200 kHz with an increment of 10 kHz were utilized to excite microbubble oscillation. Prominent microstreaming induced by microbubble oscillation was observed at 107 kHz and thus the exciting frequency of 107 kHz was chosen for all the experiments. An air bubble was generated and trapped at the rectangular microcavity when the PBS solution was injected into the microchannel due to the surface tension. Polystyrene (PS) particles with a diameter of 2 μm were chosen as tracer particles to characterize the microstreaming induced by the oscillating microbubble [23,34]. When an input voltage of 144 Vpp was applied to the PZT, the trapped air bubble started to oscillate immediately and vigorously. Figure 2 shows that the PS particles follow two symmetric near-ellipsoidal trajectories. Furthermore, the flow velocity at various positions within the flow filed was significantly different.

**Figure 3:**
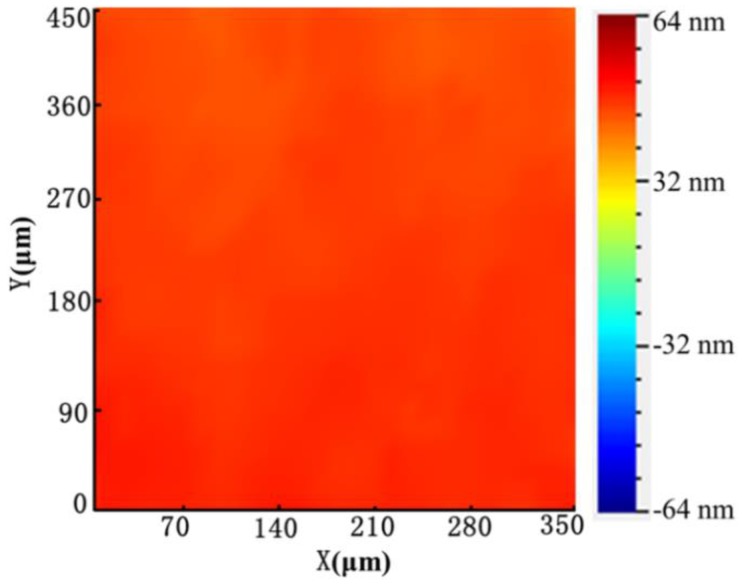
The vibration amplitude of the glass substrate. It can be seen that the vibration amplitude of the position, where the PDMS channel is located, is relatively uniform.

When input voltage is applied to PZT, the cells can be trapped on the surface of oscillating bubbles within seconds, as shown in Video S1. The trapping mechanism was mainly based on two primary forces applied to cells induced by the vibrated bubble. The two primary forces included microstreaming-generated drag force and the secondary acoustic radiation force induced by the oscillating bubble. Additionally, from Figure 3, we obtain that the ultrasound energy is uniform on the microchannel by LDV.

**Figure 4:**
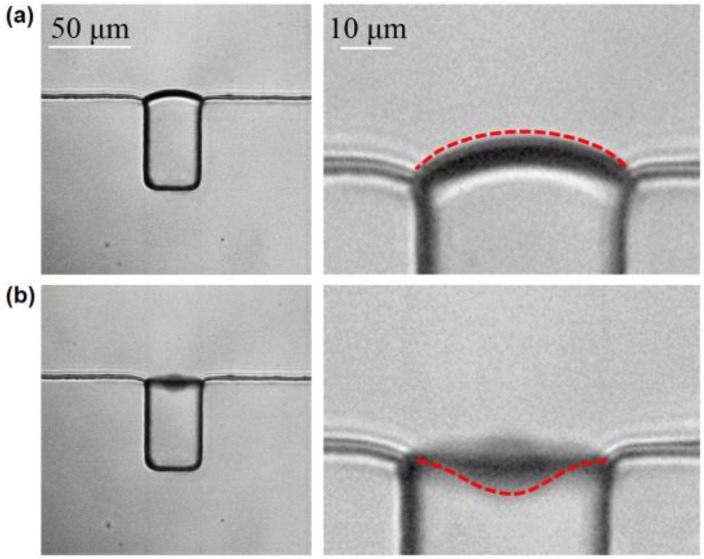
The membrane deformation of the oscillating bubble. (**a**) No deformation of the oscillating microbubble is observed without ultrasound irradiation. (**b**) The deformation of the bubble membrane is approximately 16.23 μm when the input voltage is 144 Vpp.

We can estimate the magnitude of shear stress for cell lysis by the following Equation [20,35,36]:

(1) S=2π32ε2(ρff3μ)12/Rb,

where $R_{b}$ ≈ 20 μm is the radius of a microbubble, f ≈ 107 kHz is the resonance frequency, $\rho_{f}$ ≈ 1148 kg·m^−3^ is the density of the phosphate buffer (PBS) solution, μ ≈ 1010 Pa·s is the relative streaming velocity of the fluid, and ε ≈ 16.23 μm is the oscillating amplitude of the microbubble. The shear stress on the MCF-7 cell was calculated to be 5.1 kPa when the input voltage was 144 Vpp, and cell deformation with an amplitude of 16.23 μm was recorded by a high-speed CCD, as shown in Figure 4.

### 3.2. The Stable Cavitation of Microbubble

**Figure 5:**
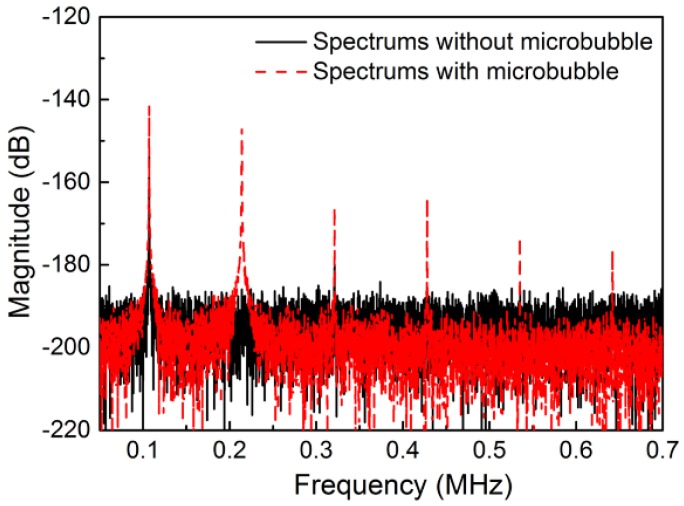
Microbubble stable cavitation. Higher harmonics are detected, illustrating that stable cavitation occurred (red dashed line). Without microbubbles, only the fundamental frequency spectrum can be detected (black line).

In the presence of microbubbles, acoustic cavitation may be the primary reason leading to cell lysis [21,35,37]. To validate whether stable cavitation occurred, a PCD system based on LDV was utilized. Without microbubbles, only the fundamental frequency of the incident ultrasound could be detected (blackline in Figure 5). However, multiple higher harmonic signals were detected in the microchannel of the cell lysis device, indicating that the stable cavitation had occurred. Moreover, there was no wide-band noise resulting from the oscillating microbubble, indicating that inertial cavitation was prevented. Additionally, optical imaging did not detect the collapse of the oscillating microbubble, which confirmed that the inertial cavitation was eliminated. Moreover, we found that when the air bubble diameter was decreased to 20 μm, the stable cavitation effects induced by the bubble oscillation were relatively small and the lysis efficiency would be decreased correspondingly. Therefore, the bubble diameter was kept at 40.8 μm for realizing the high efficiency of cell lysis.

**Figure 6:**
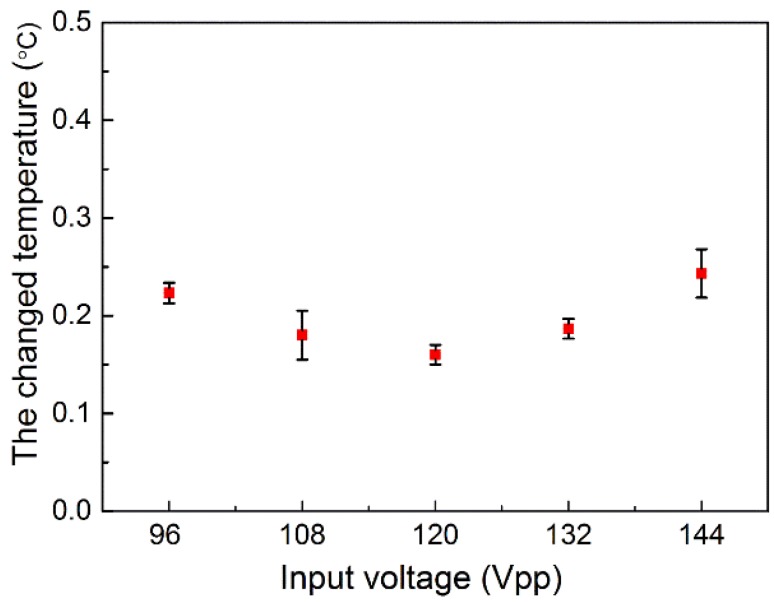
The surface temperature of the PDMS for different input electronic powers. There is no significant difference for input voltages of 96, 108, 120,132, and 144 Vpp. The temperature elevation is as small as 0.3 °C.

**Figure 7:**
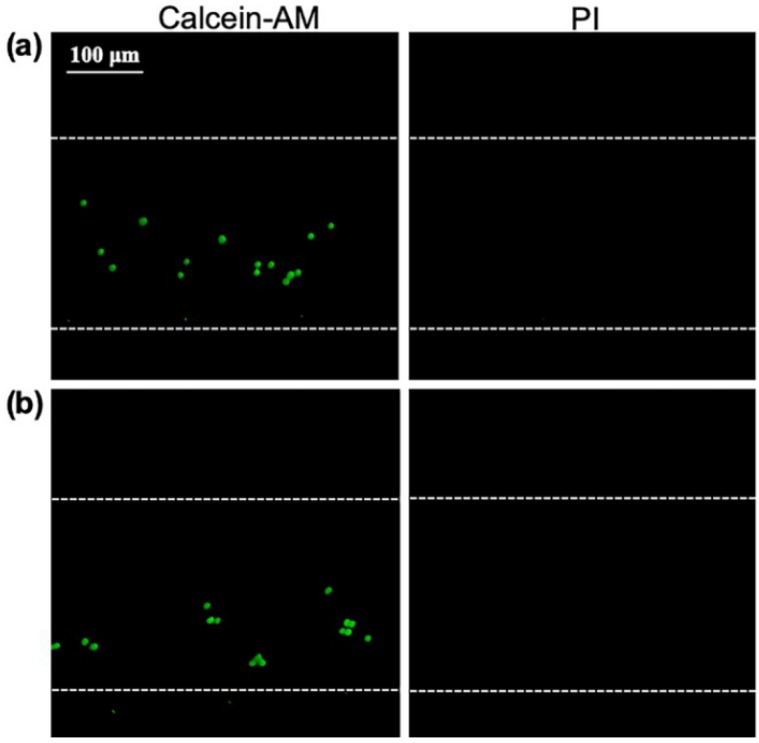
The extent of cell death. (**a**) The fluorescence images of MCF-7 after 3 min sonication, where the cell is excited by the PZT at frequency of 400 kHz. (**b**) The fluorescence images of MCF-7 with no acoustic excitation.

Further experiments were conducted to investigate whether the thermal effects induced by the PZT influenced cell lysis. The temperature of the PDMS was recorded as equal to the room temperature of 28.5 °C, when the input voltage was 96, 108, 120,132, and 144 Vpp, with a sonication time of 3 min. Figure 6 shows that the change in temperature was approximately 0.3 °C, which should have little impact on the cell lysis. Moreover, the cells stained with Calcein-AM and PI were injected into the channel and the cells were excited by the PZT, with the resonant frequency of 400 kHz, where there was no oscillation for bubble. The input voltage was 144 Vpp and the ultrasonic time was 3 min. Additionally, the negative control group was also performed with no acoustic excitation. Figure 7 shows the red fluorescence of PI cannot be observed after 3 min in ultrasonic group or negative control group, indicating that the cells in the microchannel were still alive and the thermal effect had no influence on cell lysis. Thus, the result suggests that the possible mechanism involved may be primarily due to a short-range volume interaction between the cell and the nearest bubbles. Moreover, the mechanism of cell lysis is mainly depended on the shear stress resulting from the individual oscillating microbubbles.

### 3.3. A Single-Cell Lysis

**Figure 8:**
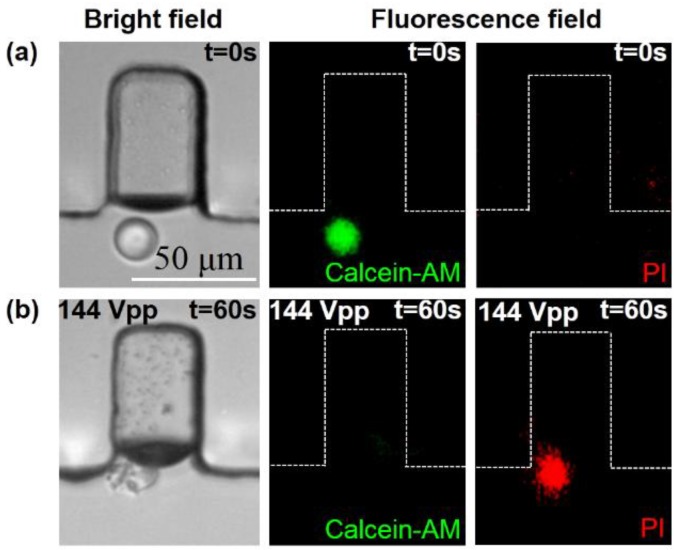
MCF-7 cell lysis. (**a**) The image series shows a single cell in the vicinity of the air bubble before the application of the ultrasound sonication. As the MCF-7 cell membrane is intact, the green fluorescence generated by the living cells is observed. (**b**) After the application of the ultrasound irradiation, the MCF-7 cell is broken into pieces, and only the red fluorescence of the PI is observed.

The MCF-7 cells suspended in the cold PBS buffer were injected into the channel at a flow rate of 0.2 μL·min^−1^ from the inlet. Prior to lysis, the MCF-7 cells were stained with Calcein-AM and PI. Calcein-AM as a marker for living cells can enter the cells when the cell membrane is intact, which generates green fluorescent light. Once the cell was lysed, the cell emitted red fluorescent light as PI crossed the membrane and bonded with the DNA. During the cell lysis process, a signal generator was used to apply the external radio frequency (RF) signal at the 107 kHz resonant frequency of the PZT, and the input voltage was 144 Vpp. As shown in Figure 8, the bright-field image shows that the cell was broken into several fragments due to the influence of the oscillating microbubble. After application of the ultrasound sonication, red fluorescence could be observed, while the green fluorescence disappeared. Figure 8 indicates that the targeted cell was lysed completely when the input voltage was 144 Vpp with a sonication time of 60 s.

### 3.4. Cell Lysis Efficiency

**Figure 9:**
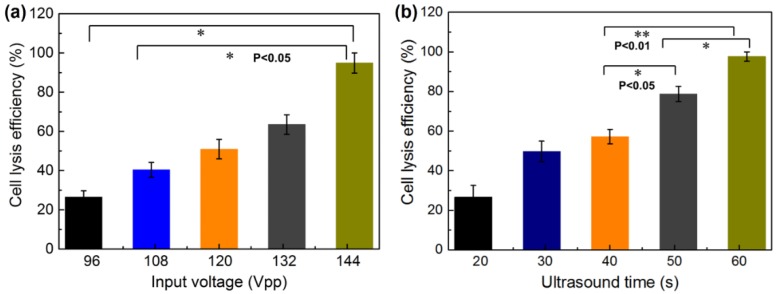
MCF-7 cell lysis efficiency. (**a**) Lysis efficiency of MCF-7 cells for different input voltages of 96, 108, 120,132, and 144 Vpp and the lysis time is 3 min. The cell lysis efficiency increases with the increment of input voltage. (**b**) MCF-7 cell lysis efficiency for ultrasonic times of 20, 30, 40, 50, and 60 s when the input voltage is 144 Vpp. The cell lysis efficiency increased with the increment of ultrasonic time.

To investigate the influence factors of the cell lysis efficiency, the MCF-7 human breast cell line was lysed with variations of the input power and ultrasonic treatment time. The lysis efficiency was defined as the percentage of PI (red) stained cells among all cells stained by Calcein-AM (green). The variation of input powers selected for the lysis of MCF-7 cells was applied to optimize the lysis power. The lysis conditions were set as follows: input voltages of 96, 108, 120,132, and 144 Vpp and ca ell lysis time of 3 min. Figure 9a shows the cell lysis efficiencies of 26.45, 40.37, 50.95, 63.49, and 98.12%, corresponding to input voltages of 96, 108, 120,132, and 144 Vpp. The lysis efficiency of cell samples increased rapidly with the increment of input power, at a cell density of 2 × 10^6^ cell·mL^−1^. Therefore, the input voltage of 144 Vpp should be the optimized input power for cell lysis.

The effect of the ultrasonic time of the PZT microchip on cell lysis efficiency was also assessed. The lysis conditions were set as follows: MCF-7 cell density of 2 × 10^6^ cells·mL^−1^ and input voltage of 144 Vpp. As shown in Figure 9b, when the treatment time changed from 10 to 60 s, the cell lysis efficiency significantly increased from 26.63% to 97.62%. When the ultrasonic time increased to 3 min, the efficiency of cell lysis was 98.12%. Prolonging the ultrasound treatment time (larger than 60 s) resulted in no significant increment in lysis efficiency when the voltage was 144 Vpp. Thus, the optimized ultrasound treatment time was 60 s.

### 3.5. DNA Agarose-Gel Electrophoresis Detection

**Figure 10:**
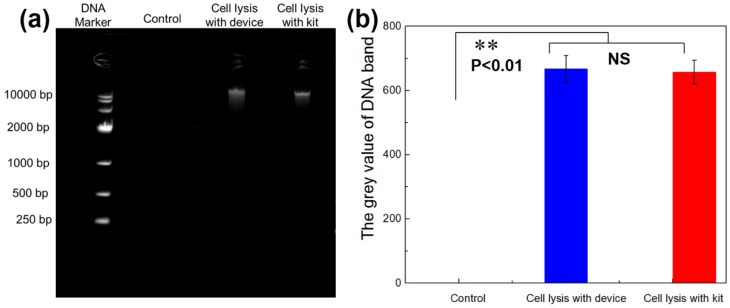
DNA gel electrophoresis of MCF-7 cells. (**a**) The control group, MCF-7 cells without ultrasound, shows no DNA band. The DNA band is observed by both the microfluidic device and commercial kit, indicating that the cells are cracked, and then the nucleic acids of the MCF-7 cells are released. (**b**) Semi-quantitative analysis of DNA production with Image J software. There is no significant difference between the results of the device and kit, but there is a significant difference compared to the control group.

As DNA is an important genetic material in cells, it is necessary to measure the amount of DNA in the lysed products for determining whether a cell is completely lysed. This has been performed using the DNA agarose-gel electrophoresis method [38,39]. The same number of cells was utilized for commercial DNA extraction kits and this lysis device to test the performance of the device. Additionally, the control group was the sample solution collected from the outlet without ultrasound sonication. Figure 10 shows that the molecular weight of DNA was close to 10,000 bp, and no significant difference was detected using this device compared to commercial DNA extraction kits for cell lysis. There was a need for the chemical commercial kit to add some enzymes to destroy the membrane skeleton, in order to release DNA, RNA or proteins for more than 15 min. The lysis device proposed in this paper only takes 1 min for cell lysis. Therefore, the consumption time of MCF-7 cell lysis for obtaining an equal amount of DNA with our device was nearly 15 times shorter than with the chemical commercial extraction DNA kit. Thus, the cell lysis device can break the cells quickly and efficiently to successfully detect the DNA contents.

## 4. Conclusions

In conclusion, we developed a cell lysis device based on a stable cavitation microbubble array. An oscillating microbubble array applied shear stress of 5.1 kPa to the targeted cell on the bubble surface, whose cell membrane was prone to lyse. Moreover, lysis efficiency could reach 97.62%. With the same concentration of cells, the consumption time of cell lysis is 1 min with our microchip device, while it takes 15min for cell lysis with the chemical commercial method. Therefore, the cell lysis consumption time is reduced by nearly 15 times in comparison to the commercial method. The advantages of the lysis device lie in the simplicity of operation, high lysis speed, and high lysis efficiency. Therefore, the lysis device constitutes a powerful tool for bio-detection, diagnosis, and biological sample pre-treatment applications.

## Supplementary Materials

The following are available online at https://www.mdpi.com/2072-666X/11/3/288/s1, Video S1: Cell trapping and releasing.
